# Synergistic Activation of *RD29A* Via Integration of Salinity Stress and Abscisic Acid in *Arabidopsis thaliana*

**DOI:** 10.1093/pcp/pcw132

**Published:** 2016-08-06

**Authors:** Sang Y. Lee, Neville J. Boon, Alex A.R. Webb, Reiko J. Tanaka

**Affiliations:** ^1^ Department of Bioengineering, Imperial College London, London SW7 2AZ, UK; ^2^ Department of Plant Sciences, University of Cambridge, Cambridge CB2 3EA, UK

**Keywords:** Combined stress, Gene expression dynamics, Mathematical modeling, Plant abiotic stresses, Signaling cross-talk

## Abstract

Plants perceive information from the surroundings and elicit appropriate molecular responses. How plants dynamically respond to combinations of external inputs is yet to be revealed, despite the detailed current knowledge of intracellular signaling pathways. We measured dynamics of *Response-to-Dehydration 29A* (*RD29A*) expression induced by single or combined NaCl and ABA treatments in *Arabidopsis thaliana. RD29A* expression in response to a combination of NaCl and ABA leads to unique dynamic behavior that cannot be explained by the sum of responses to individual NaCl and ABA. To explore the potential mechanisms responsible for the observed synergistic response, we developed a mathematical model of the DREB2 and AREB pathways based on existing knowledge, where NaCl and ABA act as the cognate inputs, respectively, and examined various system structures with cross-input modulation, where non-cognate input affects expression of the genes involved in adjacent signaling pathways. The results from the analysis of system structures, combined with the insights from microarray expression profiles and model-guided experiments, predicted that synergistic activation of *RD29A* originates from enhancement of DREB2 activity by ABA. Our analysis of *RD29A* expression profiles demonstrates that a simple mathematical model can be used to extract information from temporal dynamics induced by combinatorial stimuli and produce experimentally testable hypotheses.

## Introduction

Plants sense external stress information and make adequate decisions to commit cellular resources toward eliciting appropriate responses. Due to their sessile lifestyle, however, plants do not possess the ability to influence the surrounding environment directly, which often results in exposure to multiple types of stresses. Knowledge of the signaling mechanisms for integrating multiple stress signals and optimal control of their molecular and physiological responses is therefore crucial for understanding how plants successfully adapt to hostile changes in their environments.

The importance of the responses to combined stress in understanding plants’ adaptation to complex environments has led to numerous efforts to characterize the effects of combining multiple stresses on physiological characteristics such as growth, and molecular changes such as gene regulation (Mahalingam 2015). This has demonstrated that plants treat stress combinations as a new environment, rather than the additive sum of individual stresses (Mittler 2006). Based on a simple binate interpretation of interactions between stresses as synergy or antagonism, the non-additive effects of numerous stress pairs on broad physiological traits such as growth and yield have been analyzed (Suzuki et al. 2014). The results suggest that most stress combinations interact synergistically, inflicting greater damage on plants in comparison with singly applied stresses: for example, drought and heat, which is one of the most commonly observed stress combinations, exacerbate the detrimental effect on photosynthetic capacity and growth (Chaves et al. 2003, Vile et al. 2012). On the other hand, several stress combinations are known to result in antagonistic interactions by either mitigating the damage or enhancing tolerance to the other stress, such as increased protection against O_3_ uptake and its associated damage by decreased stomatal conductance caused by drought stress (Pääkkönen et al. 1998, Biswas et al. 2011).

It is therefore necessary to determine the molecular signaling mechanisms that are responsible for non-additive behaviors arising from combination of multiple stresses. The progress made in identifying signaling networks activated by a single stimulus provides only partial insight into the interaction mechanisms because the current models of stress signaling pathways have limited explanatory power beyond the single stress conditions from which they were constructed. For instance, it is currently not possible to predict the transcriptional response to combinatorial stress based on current understanding of the role of promoter *cis*-regulatory elements determined from single stress investigations, as many transcripts have counter-intuitive behaviors such as cancellation of responses or reversal of regulatory outcomes (Rasmussen et al. 2013, Johnson et al. 2014). The analysis of existing data sets of combinatorial stress treatments is complex because there is variation in the transcriptional response in time, the order in which the pair of stresses were applied and the developmental stage of the plants (Mahalingam 2015).

In this study, we aim to address the challenge of reconstructing regulatory network models with explanatory powers for combinatorial stresses by reducing the problem to one combination of stresses and a single transcriptional response. We investigated the response of *Arabidopsis thaliana* to NaCl stress in combination with ABA signaling. ABA acts as a hormonal signal for many different stress types, such as drought, salt, cold, pathogenic and UV irradiation stress (Finkelstein 2013). Because salt stress can trigger some responses from ABA signaling by induction of de novo ABA biosynthesis (Xiong and Zhu 2003), studying how exogenous ABA signaling interacts with salt stress provides a foundation for understanding the molecular mechanisms of interaction between salinity and other types of stresses that use ABA as a hormonal messenger. Previous studies demonstrate that the ABA and salt stress do not act independently to regulate gene expression. Xiong et al. (1999) reported that various combinations of NaCl, dehydration, ABA and cold treatments led to synergistic activation of *Responsive-to-Dehydration 29A* (*RD29A*), which encodes a 78 kDa hydrophilic protein (Yamaguchi-Shinozaki et al. 1993) of unknown function (Msanne et al. 2011). Given that the synergy between NaCl and ABA in inducing *RD29A* expression reported in these studies is deduced from single time-point measurements, however, it is still unclear how the combined NaCl and ABA stimuli affect temporal dynamics of *RD29A* expression.

By generating temporal profiles of *RD29A* transcript abundance in response to ABA and NaCl, singularly and in combination, we investigated whether the existing model of the ABA signaling pathway and ABA-independent salt stress signaling pathway upstream of *RD29A* can explain the induction of *RD29A* by combinatorial treatments. Using these data, we constructed a mathematical model of the *RD29A* regulatory network, and explored whether structural modifications in the proposed mathematical model are required to reproduce the full set of experimental data. The result of our combined experimental and theoretical approach subsequently generated novel predictions regarding where the observed synergistic effect could originate in the underlying regulatory network structure, providing a theoretical basis for further experimentation.

## Results

### Characteristic features of experimentally observed *RD29A* expression dynamics under various combinations of NaCl and ABA

Relative *RD29A* transcript abundance from 5- to 6-week-old Col-0 seedlings was measured in the absence of NaCl stress and ABA inputs (H_2_O only, control), and after different durations of treatment (0, 0.5, 1, 2, 3 and 5 h after initial exposure to input) induced by NaCl only (150 and 300 mM), ABA only (50 and 100 µM), and combination of both at full-strength (300 mM NaCl + 100 µM ABA) and at half-strength (150 mM NaCl + 50 µM ABA). The data show the relative fold increase in *RD29A* transcript level with respect to the basal level at the start of experiments (0 h). Since *RD29A* expression was found to fluctuate over time even in the absence of the inputs (Supplementary Fig. S1a) due to intrinsic circadian oscillation (Dodd et al. 2006), we normalized each of the input-induced profiles by the unstressed profile to reveal the dynamics of *RD29A* expression induced only by the treatments (see the Materials and Methods).

**Fig. 1:**
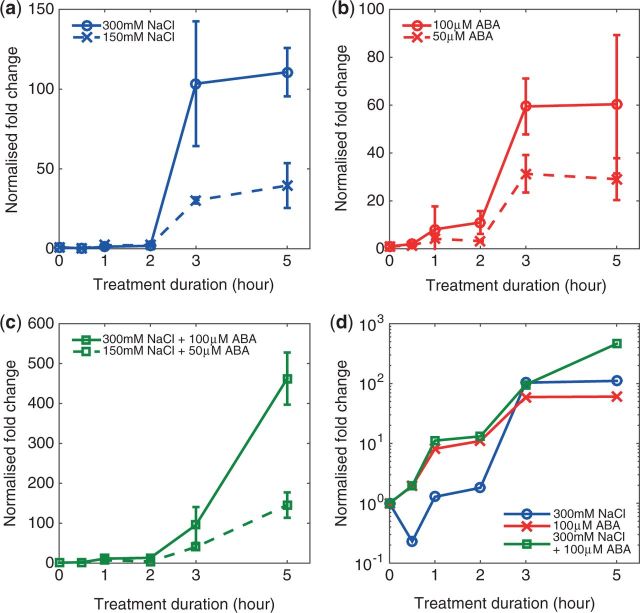
Experimentally observed *RD29A* expression profiles under (a) single NaCl treatment at full- and half-strength, (b) single ABA treatment at full- and half-strength, and (c) combined NaCl and ABA treatment at full- and half-strength. Error bars represent ± SD. (d) Comparison of the *RD29A* expression profiles induced by single and combined inputs at full-strength. To better visualize how the profiles compare during the early phase of expression (<2 h), the vertical axis was converted to logarithmic scale.

The resulting, circadian-free *RD29A* expression profiles induced by various treatment conditions (Fig. 1) showed three notable features.

*Feature 1: accumulation of RD29A transcript occurs in two phases. RD29A* expression profiles under all treatment conditions consist of two distinct phases. During the early phase (≤2 h of treatment), only a small increase of expression is observed with a negligible increase induced by 300 mM NaCl stress and an approximately 10-fold increase induced by 100 µM ABA (Fig. 1a, b). Transcript abundance during the late phase (>2 h of treatment) is significantly greater than that in the early phase, where 300 mM NaCl induces up to a 110-fold increase in transcript abundance, while 100 µM ABA induces up to a 60-fold increase (Fig. 1a, b). Combined stimulation resulted in much larger increases in *RD29A* transcript abundance, up to 460-fold by the combined NaCl and ABA inputs at full-strength, and up to 150-fold at half-strength (Fig. 1c, d). Abrupt changes in transcript abundance are observed under all treatment conditions between 2 and 3 h post-stress, suggesting that the main production of *RD29A* transcripts initiates mainly after 2 h of stress exposure (Fig. 1).

*Feature 2: strength of stress input only affects the magnitude of fold increase in RD29A expression, not its dynamics. *Comparison of *RD29A* expression profiles induced by full- (Fig. 1, solid lines) and half-strength inputs (Fig. 1, dashed lines) shows that a higher concentration of input leads to a stronger induction of *RD29A* transcription. However, the dynamics of *RD29A* expression is unaffected, as changes in the strength of input cause fold change evenly across all data points in the time-course profile. For example, halving of the ABA concentration reduces the expression fold change by approximately half across all data points, such that both full- and half-strength expression profiles show the same qualitative dynamics.

*Feature 3: there are steady increases in transcript abundance from 3 h under combined stress condition*s*, but not under single stress condition*s*. *The main qualitative difference between the *RD29A* expression profiles induced by single and combined treatments is observed from 3 h of stress treatment onwards. Such a difference is exemplified by the results of two-sample *t*-test (α = 0.01) between the measurement samples at 3 and 5 h of stress treatment (Supplementary Table S1). While no significant differences between transcript abundance at those two time points were observed under single input conditions, significant differences were observed under combined inputs both at full-strength (*P* = 0.007) and half-strength (*P* = 0.005). Such a difference suggests that there is a continued net production of transcript under combined stresses, leading to higher levels of transcript abundance at 5 h of stress than sums of transcript levels in response to individual NaCl and ABA stress (Fig. 1c, d). We call such an increase in expression specific to combined NaCl + ABA treatment the ‘synergistic effect’.

### Mathematical model of *RD29A* regulatory system

**Fig. 2:**
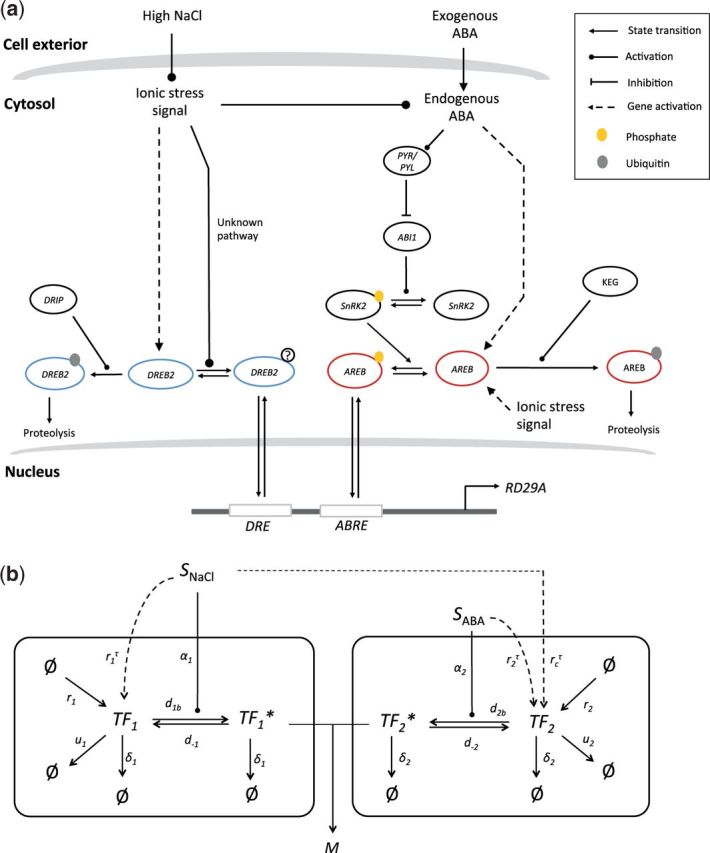
(a) A schematic diagram summarizing the current understanding of the DREB2 (left) and the AREB pathway (right). NaCl stress and ABA increase the amount of transcriptionally active DREB2 and AREB via two routes: (i) an enzymatic cascade leading to post-translational modification of the TF proteins (phosphorylation cascade involving PP2C/SnRK2 for AREB, unknown mechanism for DREB2), and (ii) induction of TF gene expression. DREB2 and AREB proteins are subject to ubiquitin-mediated proteolysis mediated by DRIP and KEG, respectively. Currently there is no evidence that DRIP and KEG activities are affected by NaCl and/or ABA. For details see main text. (b) The proposed mathematical model of the DREB2 (*TF*_1_) and AREB (*TF*_2_) pathways. The same arrowheads defined in (a) are used to describe different types of biological processes. Asterisks (*) denote the post-translationally modified form of the TF protein. Dashed arrows indicate the kinetic processes associated with time delay. Arrows originating from null sets (ø) denote de novo production of proteins, while arrows pointing towards null sets represent degradation of proteins. Model parameters for kinetic rates of the signaling processes are shown next to the corresponding arrows.

In order to investigate the origin of the three features described above, we developed a mathematical model of the *RD29A* regulatory system. The current understanding of the *RD29A* regulatory system is summarized in Fig. 2a.

Inducibility of *RD29A* expression by ABA and salt stress is conferred by the ABA-responsive element (ABRE) and the dehydration responsive element (DRE) in the promoter (Nakashima et al. 2009), which are targeted by ABRE-binding (AREB)/ABRE-binding factor (ABF) and DRE binding-2 (DREB2) proteins, respectively. AREB/ABF consists of transcription factors (TFs) belonging to the basic leucine zipper (bZIP) family that facilitate gene regulation in response to drought and salinity stress, targeting the genes containing ABRE in their upstream promoter regions (Fujita et al. 2005). The DREB2 is a subclass of DREB transcription factors that belong to the APETALA2 (AP2)/ethylene-responsive element-binding (EREBP) family, mainly regulating the genes responsive to drought and salt (Nakashima et al. 2000, Sakuma et al. 2006). Because numerous isoforms of AREB and DREB2 genes located at multiple loci are known to be functionally redundant (Gilmour et al. 2004, Yoshida et al. 2014), we do not distinguish those isoforms in our model.

The design principles of the AREB and the DREB pathways are understood reasonably well in single stress settings, with detailed functional understanding of their components and the signaling processes they regulate. NaCl stress and ABA trigger post-translational modification of their corresponding TF proteins through enzymatic cascades. For instance, AREB requires phosphorylation prior to binding ABRE (Uno et al. 2000, Furihata et al. 2006). The pathway leading to post-translational AREB activation is well understood; upon binding of ABA, pyrobactin-like/pyrobactin (PYR/PYL) receptor sequestrates the activity of protein phosphatase 2Cs (PP2Cs) such as ABI1 (Park et al. 2009), which prevents auto-phosphorylation of SNF-related kinase 2 (SnRK2) in the absence of ABA. Accumulation of phosphorylated SnRK2 subsequently leads to AREB phosphorylation (Fujita et al. 2005). DREB2 is also considered to require post-translational activation prior to binding to DRE, as its transcriptional activity of DREB2 is not proportional to its abundance (Sakuma et al. 2006, Morimoto et al. 2013). The exact nature and mechanism of DREB2 post-translational activation is currently unknown.

NaCl stress and ABA also control TF protein concentration, by increasing the expression of the TF genes directly. DREB2 genes are induced by NaCl and osmotic stresses but not significantly by ABA (Liu et al. 1998). All AREB genes are inducible by the presence of exogenous ABA as well as NaCl stress (Uno et al. 2000, Fujita et al. 2005). We assume that induction of TF gene expression increases the TF protein population. Combined with the stress cues leading to post-translational activation of the TF proteins, such an increase in the inactive TF protein population results in stronger induction of *RD29A* expression. TF protein concentration is negatively regulated by ubiquitin-dependent proteolysis mediated by RING domain E3 ubiquitin ligases such as DREB2-onteracting proteins (DRIPs) for DREB2 (Qin et al. 2008) and KEEP ON GOING (KEG) for AREB (Chen et al. 2013). Those E3 ubiquitin ligases are responsible for keeping the level of stress-inducible TFs low in the absence of stresses to avoid unwanted expression of stress response genes and waste of cellular resources in maintaining the TF population.

The schematic diagram for our mathematical model of the *RD29A* regulatory system is shown in Fig. 2b. Note that we obtained a simplified description of the core processes that lead to control of the *RD29A* level from the full model of the *RD29A* regulatory network (Fig. 2a). For instance, the simplified mathematical model ignores de novo ABA biosynthesis induced by salt stress based on the difference in the amount of ABA synthesized and the amount imported from the exogenous pool. DREB2 proteins are represented by a single variable *TF*_1_ (where TF denotes transcription factor) and AREB proteins by *TF*_2_. The stress inputs *S*_NaCl_ and *S*_ABA_ induce transcription of the *RD29A* gene through production of *TF_i_* (*i* = 1, 2), which denotes the inactive form of TF proteins, and conversion of *TF_i_* to the post-translationally activated form, TF_i_*. The effect of *S*_NaCl_ and *S*_ABA_ on *TF_i_* activation is set to be linear because a more complex, non-linear relationship does not provide any further advantage in describing the apparent linear dependence seen from the experimental data. Note that assuming linear dependence of TF activity on the input signals led to absorption of several known steps of signal transduction, such as the phosphorylation cascade involving SnRK2 and DRIP upstream of AREB, into a single linear kinetic process. Production of functional TF proteins from induction of their genes is simply described as delayed processes in the model. The model output, *M*, which denotes a relative increase of *RD29A* transcript from the start of stress treatment, is described by an algebraic sum of *TF*_1_*(*t*) and *TF*_2_*(*t*) at given time *t.* The series of simplifying assumptions adopted to acquire all of these simplifications are described in the Materials and Methods in more detail.

The model equations representing Fig. 2b are also shown in the Materials and Methods, with the full list of model parameters and their biological meanings provided in Table 1. The model consists of 16 parameters in total, among which five parameters were fixed based upon the assumptions derived from our experimental data or the existing evidence in the literature. The values of the remaining parameters were estimated by parameter optimization (Materials and Methods). The resulting nominal parameter sets are shown in Supplementary Table S2.

**Table 1:** Description of model parameters*^a^*

Pathway	Name	Biological process	Method of determination
*TF*_1_ (DREB2)	*r*_1_	Basal *TF*_1_ production rate	Fixed (Liu et al. 1998, Sakuma et al. 2006)
	δ_1_	Natural decay rate (for both *TF*_1_ and *TF*_1_*)	Fixed (Pratt et al. 2002)
	*d*_1*b*_	Basal *TF*_1_ activation rate	Fixed (Sakuma et al. 2006)
	*r*_1_^τ^	*S*_NaCl_-induced *TF*_1_ production rate	Parameter optimization
	α_1_	*S*_NaCl_-induced *TF*_1_ activation rate	Parameter optimization
	*d*_–1_	Basal *TF*_1_* deactivation rate	Parameter optimization
	*u*_1_	*TF*_1_ ubiquitination rate	Parameter optimiszation
*TF*_2 _(AREB)	δ_2_	Natural decay rate (for both *TF*_2_ and *TF*_2_*)	Fixed (Pratt et al. 2002)
	*d*_2*b*_	Basal *TF*_2_ activation rate	Parameter optimization
	*r*_2_	Basal *TF*_2_ production rate	Parameter optimization
	*r*_2_^τ^	*S*_ABA_-induced *TF*_2_ production rate	Parameter optimization
	α_2_	*S*_ABA_-induced *TF*_2_ activation rate	Parameter optimization
	*d*_–2_	Basal *TF*_2_* deactivation rate	Parameter optimization
	*u*_2_	*TF*_2_ ubiquitination rate	Parameter optimization
	*r_c_* ^τ^	*S*_NaCl_-induced *TF*_2_ production rate (for further production of *TF*_2_ via *C*_2_)	Parameter optimization
Both	τ	Time delay before TF production	Fixed (our experimental data)

*^a^* All parameters, except τ that is measured in hours, have the unit of h^–1^.

### The proposed mathematical model reproduces the experimentally observed *RD29A* expression profiles induced by single NaCl and ABA, but not by their combinations

**Fig. 3:**
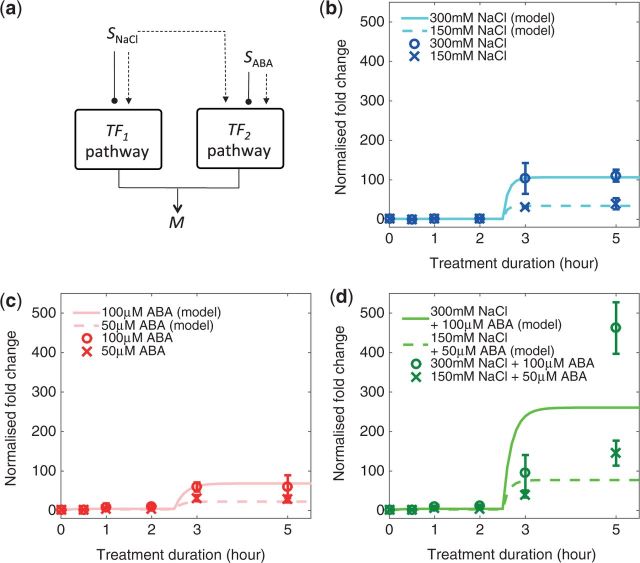
Comparison of the model solutions with the experimental data. (a) A simplified model diagram only showing the top-down regulatory cues from *S*_NaCl_ and *S*_ABA_ inputs. (b) Model with the experimentally observed *RD29A* expression fold change (circle = full-strength, cross = half-strength) under single NaCl treatment, (c) single ABA treatment and (d) combined NaCl and ABA treatment.

We assessed whether the proposed mathematical model can simultaneously reproduce the three features observed from the *RD29A* expression profiles under various treatment conditions by comparing the experimental data and the model predictions (Fig. 3).

The proposed model reproduces Feature 1, the distinct biphasic profiles of treatment-induced *RD29A* expression profiles. The model explains the negligible increase in *RD29A* expression during the early phase of NaCl treatments with the negligible expression of DREB2 genes (*r*_1_ ≈ 0 h^–1^) in unstressed conditions (Liu et al. 1998), which subsequently leads to absence of DREB2 proteins even though the enzymatic cascades leading to post-translational activation of TFs are immediately switched on after introduction of the stress. A small, 10-fold increase in *RD29A* expression during the early phase of ABA treatments is explained by weak constitutive AREB gene expression (*r*_2_ > 0 h^–1^) observed in unstressed conditions (Fujita et al. 2005), which leads to a small amount of AREB protein available for immediate activation after introduction of ABA. During the late phase, the strong increase in *RD29A* expression observed from both single NaCl and ABA treatments occurs due to further production of TF proteins via induction of TF genes (*r*_1_^τ^, *r*_2_^τ^ and *r_c_*^τ^) with the intrinsic time delay. Since such transition in the model behavior is observed between 2 and 3 h of treatment, the time delay associated with further production of TF is assumed to be fixed (τ = 2.5 h).

The model also reproduces Feature 2, where varying the strength of stress input only affects the magnitude of expression without introducing qualitative changes to the time-course profiles. The ability of our model to reproduce this feature suggests that our previous assumption that the strength of the external stimulus affects the rate of TF post-translational activation in a linear fashion is appropriate, given the limited amount of data available.

Feature 3, the steady accumulation of *RD29A* transcript from 3 h of combined stress response, is not reproduced by the model. The model can only describe the dynamics of the combinatorially induced *RD29A* expression profile as the sum of the dynamics of singly induced profiles (Fig. 3d) because there are no non-linear interactions between the two pathways, which can act as the potential source of the synergistic effect. Given that model formulation based on the literature fails to capture the greater than additive interactions between the stresses, the model must be modified in order to capture the greater than additive expression upon combination of two stresses.

### Synergistic effects originate from the selective enhancement of either DREB2 or AREB pathway

From the comparison between the experimental data and the model above, we proposed that the synergistic effect observed from the responses to combined NaCl and ABA originates from interaction between the two stimuli, leading to ‘cross-input modulation’ in the DREB2 and AREB pathways.

**Fig. 4:**
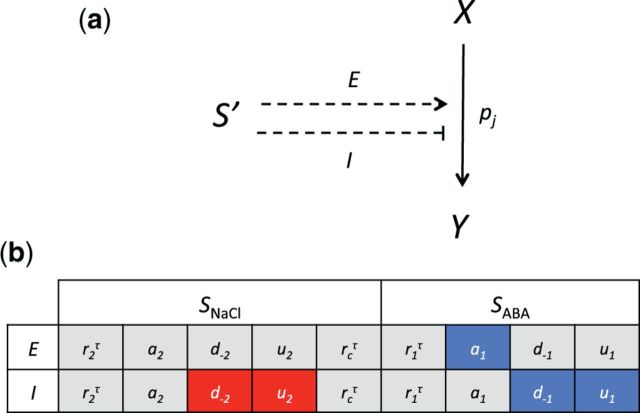
Cross-input modulation within the *RD29A* regulatory system. (a) A cross-input modulation is defined as modulation of a signaling process by the rate *p_j_*, by the adjacent, non-cognate input (*S*′). Regulatory outcome of the cross-input modulation can be by either enhancement (*E*) or inhibition (*I*) of *p_j_.* We assume that cross-input modulation is delayed by τ, hence the use of dashed lines. (b) Outline of all 18 possible system structures, organized by regulatory outcome (*E* or *I*) and the non-cognate input (*S*_NaCl_ or *S*_ABA_). The five system structures that reproduce the observed synergistic effect are highlighted in color (red = *S*_NaCl_ modulates the *TF*_2_ pathway; blue = *S*_ABA_ modulates the *TF*_1_ pathway).

We defined a cross-input modulation as a regulatory cue produced by the non-cognate input signal (*S*′), leading to enhancement (*E*) or inhibition (*I*) of the kinetic rates associated with the targeted signaling process (Fig. 4a). Biologically, cross-input modulation can occur from cross-talk interaction between two signaling pathways via shared components of the two pathways, or direct regulatory interaction between the components of the two pathways. Alternatively, cross-input modulation can also occur via a third independent pathway that utilizes none of the signaling components of the two signaling pathways, but still connects the non-cognate input signal to the affected signaling process. Although there is currently a limited amount of information to distinguish which of these mechanisms is at work, implementing cross-input modulation allows us to describe different regulatory outcomes of a cross-talk interaction or a hidden third pathway on the processes regulated by combined NaCl and ABA inputs.

Given that the synergistic effect only occurs during the late phase of expression (Fig. 1c), we assume that a cross-input modulation, which is responsible for the synergistic effect, is delayed by τ. The *RD29A* regulatory system contains nine signaling processes (*r*_2_^τ^, α_2_, *d*_–2_, *u*_2_, *r*_1_^τ^, α_1_, *d*_–1_, *u*_1_ and *r_c_*^τ^) that can form cross-input modulation. The other seven processes (*r*_1_, *r*_2_, *d*_1_*_b_*, *d*_2_*_b_*, δ_1_, δ_2_ and τ), which include basal TF production/activation rates and TF natural degradation rates, are stress independent by definition and cannot form cross-input modulation. Implementing enhancement or inhibition for each of these nine processes led to 18 modified system structures (Fig. 4b).

**Fig. 5:**
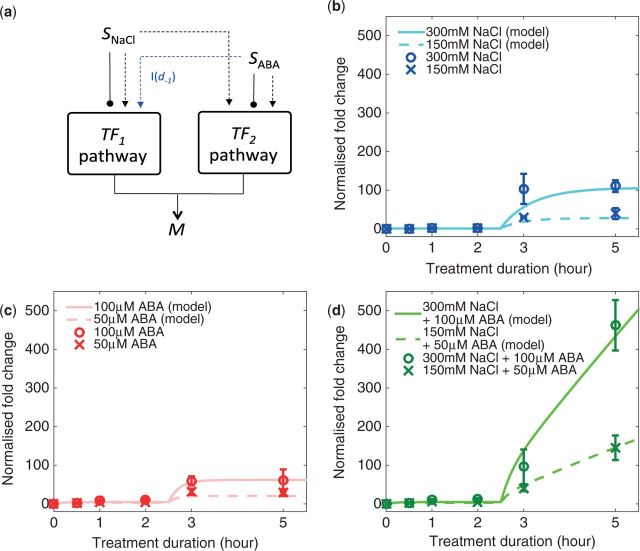
Comparison of the simulation results from the system structure *I*(*d*_–1_) (a), with the experimentally observed *RD29A* expression fold change (circle = full-strength, cross = half-strength) under (b) single NaCl treatment, (c) single ABA treatment and (d) combined NaCl and ABA treatment.

The capability of each system structure to reproduce the observed synergistic effect was assessed based on how well the model fits the combined stress response data, after independently fitting each of the other 18 system structures to the data. See Equation 9 in the Materials and Methods for the design of the objective function minimized during parameter optimization. To select the structure that can reproduce the combined stress response data, we ranked the 18 system structures according to their residual sum of squares calculated from the optimal parameter set. See Equation 10 in the Materials and Methods for the corresponding definition of residual sum of squares. We found five system structures that qualitatively reproduce the synergistic effect, as well as all other experimentally observed features (Fig. 4b). The model implementing system structure *I*(*d*_–1_) fits the data best, reproducing all three observed features (Fig. 5; see Supplementary Fig. S3 for the time-course simulations for the other four system structures). The five identified system structures show a common topological feature, where the non-cognate stress input enhances the production of the post-translationally active form of TF, denoted with TF_i_* in the model. In system structure *I*(*d*_–1_), for example, *S*_ABA_ enhances accumulation of *TF*_1_* by attenuating the rate of its post-translational deactivation. In *I*(*u*_2_), *S*_NaCl_, indirectly increases *TF*_2_* by attenuating degradation of *TF*_2_, which results in an increased net forward conversion rate into *TF*_2_*. *E*(α_1_) is also a valid form of cross-input modulation because it enhances the post-translational processing of *TF*_1_ into *TF*_1_*. Thus, the results suggest that selective enhancement of either the DREB2 or the AREB pathway may account for the synergistic effect observed from the experimental data.

The remaining 13 system structures cannot reproduce the synergistic effect qualitatively, no matter how the parameters of the original *RD29A* regulatory system models and cross-input modulation are chosen (Supplementary Fig. S4). These fail to reproduce the synergistic effect because they do not lead to selective enhancement of either pathway. For example, cross-input modulation in some system structures such as *E*(*d*_–1_) or *I*(α_2_) decreases the amount of TF_i_*, leading to attenuation of the targeted pathway instead of enhancement. Modulating production of TF proteins via gene induction (*r*_1_^τ^, *r*_2_^τ^ and *r_c_*^τ^) also does not lead to effective enhancement of the selected pathway because it increases the population of *TF_i_*, only influencing the magnitude of *RD29A* expression at steady state. Notably, inability of the structure *E*(*r*_1_^τ^) in reproducing the synergistic effect suggests that an increased rate of DREB2 production from ABA, proposed from reduced DREB2 expression upon ABA deficiency (Kim et al. 2011), is not responsible for the synergistic effect observed in response to combined NaCl and ABA treatment from our data.

### Feasibility of the identified system structures assessed from the analysis of microarray data sets

To investigate further the feasibility of the mechanisms of cross-input modulation predicted from five identified system structures *I*(*d*_–1_), *I*(*u*_1_), *E*(α_1_), *I*(*u*_2_) and *I*(*d*_–2_), we examined expression profiles of the genes that are known to mediate the five processes modulated by the cross-input (*d*_–1_, *u*_1_, α_1_, *u*_2_ and *d*_–2_) from two publicly available transcriptome-wide cDNA microarray data sets (Kreps et al. 2002, Kilian et al. 2007). Because the synergistic effect is only observed during the late phase of *RD29A* expression (Fig. 1d), we assumed that the proposed cross-input modulation is achieved by regulation of the gene responsible for the targeted signaling process. In the light of the possiblity that the targeted processes are faciliated by more than one gene, we also assumed that the selected genes are the main regulators of the affected processes in order to narrow down the list of system structures to be experimentally tested further (Table 2).

**Table 2:** Comparison of the identified system structures with cDNA microarray data sets

Viable system structures reproducing the synergistic effect			Evidence for cross-input modulation within the existing experimental data set			
Type	Name	Proposed mechanism	Candidate gene (locus)	Molecular function	Expression profiles from cDNA microarray data sets	
					Kreps et al. (2002)	Killian et al. (2007)
Enhancement of DREB2 outputs by ABA	*I*(*u*_1_)	ABA inhibits DREB2 ubiquitination (*u*_1_)	*DRIP1* (At1g06770)	E3 ubiquitin ligase (Qin et al. 2008)	Data not available	Expression independent of abiotic stress (NaCl, drought, osmotic stresses)
			*DRIP2*			
			(At2g30580)			
	*E*(α_1_)	ABA enhances DREB2 post-translational activation (α_1_)	Unknown	N/A	N/A	N/A
	*I*(*d*_–1_)	ABA inhibits post-translational deactivation of active DREB2 (*d*_–1_)	Unknown			
Attenuation of AREB outputs by NaCl	*I*(*u*_2_)	NaCl inhibits AREB ubiquitination (*u*_2_)	*KEG* (At5g13530)	E3 ubiquitin ligase (Chen et al. 2013)	Data not available	Expression independent to abiotic stress (NaCl, drought, osmotic stresses)
	*I*(*d*_–2_)	NaCl inhibits phospho-AREB dephosphorylation (*d*_–2_)	*AHG3* (At3g11410)	Protein phosphatase 2C (Lynch et al. 2012)	Expression up-regulated by NaCl	Expression up-regulated by NaCl

Suppression of DREB2 degradation in *I*(*u*_1_) is not supported by the expression profiles observed from both data sets because expression of DRIP, an E3 ubiquitin ligase responsible for targeted proteolysis of DREB2, appears independent of various abiotic stresses including ABA (Kilian et al. 2007). The expression profile of KEG obtained from one data set (Kilian et al. 2007) shows independence of NaCl stress, which contradicts attenuation of the AREB pathway claimed by *I*(*u*_2_). Both data sets contradict *I*(*d*_–2_) by showing that expression of AHG3, a gene encoding ABI-clade phosphatase (Lynch et al. 2012), is up-regulated in the presence of NaCl stress. Note that this observation does not prove that cross-input modulation of opposite regulatory outcome, i.e. *E*(*d*_–2_), exists because ABA is known to inhibit the protein activity of AHG3 strongly (Antoni et al. 2012).

Consequently, two system structures, *E*(α_2_) where ABA enhances DREB2 post-translational activation and *I*(*d*_–1_) where ABA attenuates post-translational deactivation of active DREB2, remain as viable system structures. The information regarding those system structures could not be extracted from the microarray data sets because the identities of the genes responsible for DREB2 post-translational modification are as yet unknown. This result suggests that ABA-induced enhancement of post-translational activation of DREB2 via *E*(α_1_) or *I*(*d*_–1_) is responsible for the observed synergistic effect.

### Feasibility of the identified system structures assessed from further experiments to validate model predictions

**Fig. 6:**
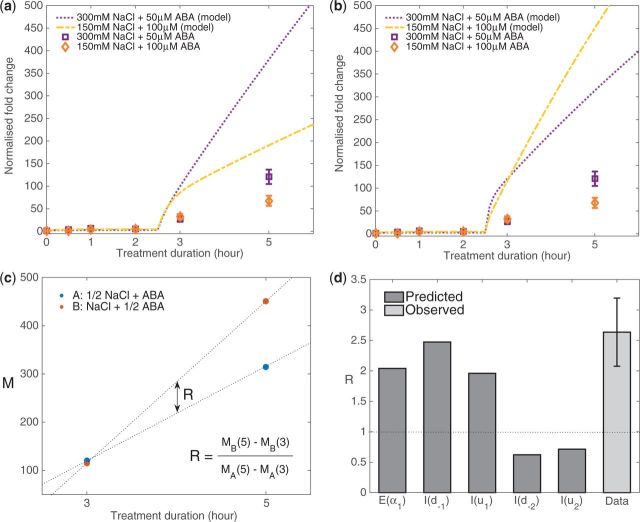
Reduction of synergistic effect upon halving the dose of one stress in a combined stress input. The responses predicted from the system structure (a) *I*(*d*_–1_) and (b) *I*(*u*_2_) are compared with the results of the subsequent model-guided experiment. (c) The synergistic effect arising from combined stress is approximated by the slope of increase in *RD29A* transcript abundance, *M*, between 3 and 5 h stress treatment. The effect of halving either stress is compared by calculating the ratio, R. (d) Comparison of the ratio R calculated from experimental data with the predicted ratios from each of the five identified system structures.

In parallel to the insights from the analysis of cDNA microarray data sets, we sought to reduce the number of possible system structures by experimentally verifying the predictions from the identified system structures upon change in treatment condition. Here, we investigated the impact on the *RD29A* expression profile from reducing the strength of one input in combined NaCl and ABA treatments. Because the identified system structures require an additional interaction to affect either DREB2 or AREB pathways, the model predicts that halving the dose of either one in combined input would result in asymmetric reduction in synergistic effect. *I*(*d*_–1_), for example, predicts that halving the concentration of NaCl stress input leads to greater reduction in synergistic effect compared with halving of ABA concentration (Fig. 6a, line), while *I*(*u*_2_) predicts the opposite (Fig. 6b, line). These predicted model solutions were obtained using the same parameter sets identified from optimization (Supplementary Table S2).

The experimentally observed *RD29A* expression profiles from combinations of NaCl and ABA stresses at unequal strength (300 mM NaCl + 50 µM ABA stress, and 150 mM NaCl + 100 µM ABA stress) show that halving NaCl input leads to greater reduction in synergistic effect (Fig. 6a, b, points). Although the results from the new experiments did not quantitatively match the predicted model solutions, they showed qualitative agreement with the predictions made from *I*(*d*_–1_). To visualize the degree of reduction in synergistic effect, we defined a ratio, R, which compares the degree of reduction in gradient of *RD29A* expression fold increase at 3–5 h post-stress triggered by halving of either NaCl or ABA stress (Fig. 6c). Comparing the R values obtained from all of the five system structures with that of the new experimental data (Fig. 6d) suggested that the predictions obtained from *E*(α_1_), *I*(*d*_–1_) and *I*(*u*_1_) qualitatively match the experimental data. Given that these three structures describe enhancement of post-translational activation of DREB2 by ABA, this result is in agreement with the hypothesis from microarray analysis that the observed synergistic effect is due to the selective enhancement of the post-translational activation of DREB2 by ABA.

## Discussion

In this article, we have used experimental and theoretical approaches in parallel to investigate how multiple external stimuli regulate temporal dynamics of gene expression, using *RD29A* expression in *Arabidopsis thaliana* as a demonstrative example. While it was already documented that combined abiotic stress signals synergistically activate *RD29A* expression (Xiong et al. 1999), our experimental data show for the first time that interaction between NaCl stress and ABA affects not only the magnitude of *RD29A* expression but also its temporal dynamics. We used the following analysis workflow to analyze the experimental data and extract the potential source of the synergistic *RD29A* activation by combined NaCl and ABA: (i) we first tested whether a simple mathematical model developed from the existing knowledge of the DREB2 and AREB pathway structures can reproduce all key qualitative features from the experimental data through parameter fitting. (ii) The model received structural modification in forms of cross-input modulation leading to a number of different system structures, and the ability of each structure to reproduce the experimental features was evaluated by fitting each structure to the experimental data again. (iii) The system structures were ranked based on quality of fit to the combined NaCl and ABA response, where synergy was observed. (iv) Finally, the top-ranking structures were subjected to further tests via analysis of the existing microarray data sets and additional hypothesis-driven experiments. Thus, the series of steps taken systematically to eliminate the model structures that are unable to reproduce the data ultimately led to the conclusion that ABA-dependent DREB2 post-translational activation is the potential source of the observed synergistic effect.

The identified interactions in the model may be tested further; time-course measurement of *RD29A* expression in mutants constitutively expressing DREB2 proteins such as 35S:DREB2A (Sakuma et al. 2006), based on *abi* (ABA-insensitive) as background is proposed. If DREB2 post-translational activity is enhanced in the presence of an ABA-dependent signaling mechanism, then deleting the activity of the ABA-dependent signaling mechanism is thought to remove the synergy in combined NaCl + ABA treatment. The reason for using the 35S:DREB2A transgene is to compensate for lower DREB2 expression in *abi* lines (Kim et al. 2011). Another possible confirmatory experiment is to obtain time-course measurements from other genes regulated by both ABRE and DRE, and investigate whether the synergy from combined NaCl and ABA treatment on *RD29A* is also observed from those genes. Promoter sequence analysis has predicted that there are 2,052 genes that contain both ABRE and DRE motifs in their non-coding regions (Mishra et al. 2014), which suggests that there could be other genes behaving in a similar manner to *RD29A.* To validate that ABA-dependent DREB2 post-translational activation is responsible for the synergy, it may also be useful to observe the genes containing either DRE or ABRE only, and then examine whether the genes containing only DRE exhibit synergy and not those containing the ABRE motif (e.g. RD29B). Given that the presence of the *cis*-regulatory element in the promoter does not always mean that the gene is regulated by the corresponding TF, however, working with a smaller subset of genes whose interaction with AREB and DREB2 proteins are well established may be necessary.

The apparent interdependence of the NaCl and ABA signals in modulating both DREB2 and AREB signaling pathways raises a further question: how does the abiotic stress system distinguish and selectively express ABRE- or DRE-controlled genes in response to single NaCl or ABA if the DREB2 and the AREB pathways are cross-modulated by both NaCl and ABA? Our model shows that such an input-specific response emerges from the activation mechanism of DREB2 and AREB, which resembles a logical ‘AND’ operator. Because activations of DREB2 and AREB require a simultaneous increase in post-translational modification and TF population through their gene expression, activation of either one mechanism is insufficient to induce their transcriptional activity. Given that the identified interaction affects only the post-translational modulation, the abiotic stress response system can avoid any unwanted outcomes of interaction between NaCl and ABA signals and produce outputs specific to the DREB2 and AREB pathways under single stress conditions.

Cross-regulation of the DREB2 and AREB pathways by NaCl and ABA has a wider implication for understanding abiotic stress response as a whole. Considering that the DRE regulon consists mostly of the genes specific to osmotic and heat stress response (Nakashima et al. 2009), we propose that selective enhancement of the DRE regulon upon combined stress conditions ensures prioritizing the immediate response to the stress without committing to long-term effects induced by ABA. This would be readily verifiable by further experiments measuring dynamics of other DRE-controlled genes. Furthermore, detailed ontology analysis of genes constituting the DRE and ABRE regulons, coupled with measurements of their expression profiles under combined NaCl and ABA, will help in understanding the physiological significance of the proposed interaction between NaCl stress and ABA inputs.

The investigative approach presented in this work may be applied to study other signaling pathways that are less explored. For example, another type of TF that is known to form ABA-dependent and ABA-dependent pathways within its family is NAC (NAM, ATAF1, 2 and CUC2) proteins which target the NAC recognition (NACR) sites (Puranik et al. 2012). The regulatory networks upstream of NAC TFs are much less studied compared with the system of our choice, but nevertheless play crucial roles in plant responses to drought, salt and ABA. It would be interesting to observe how abiotic stress such as salt and dehydration stress combined with exogenous ABA affect expression profiles of the genes controlled by NACR, and as activities of NAC TFs are also known be controlled by two-pronged regulation of the amount of active TF and mRNA quantity, a model similar to that of the DREB2 and AREB pathways can be applied and maintain some predictive power over the qualitative behavior of the system upon changes in combined stress inputs. However, whether the model based on the core structure of the NAC networks bearing resemblance to the *RD29A* regulatory system is sufficient to describe the dynamic behaviors observed from NACR-controlled genes under combined salt stress and ABA remains to be seen. Although our proposed model was able to reproduce the experimentally observed features from *RD29A* expression dynamics qualitatively with as few parameters as possible, the issue of underfitting must be considered carefully if our model is to be applied to study the effect of stress input combinations on other genes.

With an increasing number of studies examining the effect of combined stresses in plant gene regulation (Rizhsky et al. 2004, Giraud et al. 2008, Zhu et al. 2010, Burgos et al. 2011, Estavillo et al. 2011, Atkinson et al. 2013, Prasch et al. 2015), the need for mechanistic models to explain the gene expression profiles observed from the resulting data sets is also becoming greater. The current understanding of the plant stress signaling network, however, provides limited explanations for gene regulation in combined stress settings, as they are mostly based on the information obtained from single stress experiments. In the light of such challenges, use of mathematical and computational models may be an effective measure to integrate data of various types, with the ability to make a robust description and prediction of molecular and physiological processes under a variety of environmental conditions. Mathematical models have so far had limited use in understanding gene regulatory responses to multiple environmental inputs except for well-defined systems such as control of stomatal opening (Beguerisse-Diaz et al. 2012). We therefore anticipate that there is much to be offered to system-level understanding of molecular and physiological responses to combined environmental stresses in plants from application of mathematical models, allowing integration of the current knowledge of individual signaling pathways for a single type of stress signal and generation of new insights that are verifiable by further hypothesis-driven experiments.

## Materials and Methods

### Quantitative measurement of RD29A expression dynamics

#### Stress treatment and sample preparation

*Arabidopsis thaliana* (ecotype Col-0) seedlings were stratified at 4°C for 48 h, followed by growth on agar plates containing Murashige–Skoog medium for 5–6 weeks at constant temperature (20°C). The seedlings were entrained with a 12 h light/1 h dark cycle (09:00 to 21:00 h in real time) during growth, illuminated under 60 µmol m^–2^ s^–1^ white light. Seedlings were then hydroponically treated under different stress conditions. Each treatment medium with varying concentrations of NaCl and ABA was prepared by dissolving an appropriate amount of NaCl and ABA in deionized water. Untreated control samples were immersed in deionized water. Three replicate samples were made from collection of several randomly selected seedlings into three Eppendorf tubes, with each sample weighing approximately 60 mg (FW) in total. The collected samples were then immediately frozen in liquid N_2_ and stored at –80°C prior to extraction of RNA. Initiation of stress treatment and sample collection occurred at the same time of day for all experiments (07:00 to 12:00 h in Zeitgeber time). Three experimental replicate samples were obtained for each treatment condition and duration.

#### Sample processing

Tissue disruption and RNA extraction were carried out using RNEasy mini kits (Qiagen). RNA integrity was verified by using a nanodrop spectrophotometer (ND-1000, Thermo Scientific Inc.); the samples with relatively high RNA yield (500–800 ng µl^–1^) and a high DNA to RNA, RNA to salt separation ratio were selected. The resulting transcriptome samples were converted into cDNA using Quantitect Reverse Transcription kits (Qiagen). During this step, the samples were diluted accordingly to give the uniform concentration of 500 ng nl^–1^, and were treated with DNase to remove any trace of genomic DNA and obtain high-quality transcriptome samples. Real-time quantitative PCR (qPCR) experiments were carried out (Rotor-gene Q cycler, Qiagen) to measure the fold changes in *RD29A* expression compared with that of a control gene, *Actin-2.* The two genes were specifically amplified using the pre-prepared primers with the following sequences: *RD29A* forward, 5′-CCGGAATCTGACGGCCGTTTA-3′; *RD29A* reverse, 5′-CCGTCGGCACATTCTGTCGAT-3′; *Actin-2* forward, 5′-TCCTCACTTTCATCAGCCG-3′ and *Actin-2* reverse, 5′-ATTGGTTGAATACATCAGCC-3′·

The reaction conditions were prepared using Rotor-gene Syber Green PCR kits. For optimal results, the reaction samples were diluted again, such that the template cDNA amount is 20 ng per reaction. Prior to the qPCR experiments, each sample was divided further into three technical replicates in order to achieve high accuracy.

#### Data analysis

Cycle time (CT) data were obtained from the resulting ﬂuorescence data of qPCR experiments by setting a threshold value (normalized fluorescence = 2.5 × 10^–3^ RFU). The CT values for *Actin-2* transcript abundance were then subtracted from that of *RD29A* transcript abundances to obtain ΔCT for each time point, *t*. We then calculated fold change = log_2_ [ΔCT(*t*)] – log_2_ [ΔCT(0)] for each of the experimental replicates. For the plot of mean fold change, see Supplementary Fig. S1. To remove the potential effect of circadian oscillation on *RD29A* expression, we subsequently calculated normalized fold change by dividing the fold change data from each experimental replicate by the mean fold change observed under unstressed control Supplementary Fig. S1a). To quantify errors, the SD was calculated for each data point after normalization. Two-sample *t*-tests between the triplicate data points obtained at 3 and 5 h for each treatment condition were conducted using the *ttest2* function in Matlab Release 2014b.

### Model equations

#### Stress input dynamics

The dynamics of intracellular salt stress signal are described by

(1) $$S_{\text{NaCl}}\left(\text{t}\right)=\{\begin{array}{cc} \frac{{\left[\text{NaCl}\right]}_{\text{ext}}}{{\left[\text{NaCl}\right]}_{\text{max}}} & if\;t>0,\\ 0 & \;if\;t\leq 0, \end{array}$$

where [NaCl]_ext_ and [NaCl]_max_ represent the external NaCl concentration and the maximum external NaCl concentration beyond which *RD29A* expression no longer increases (300 mM) based on the data from Xiong et al. (1999). Thus, *S*_NaCl_ is a variable ranging from 0 to 1, representing the strength of salt stress. The dynamics of salt input are described by a piecewise function to mimic the abrupt increase in salt concentration occurring at the start of treatment (*t* = 0).

The dynamics of endogenous ABA are described by

(2) $$S_{\text{ABA}}\left(\text{t}\right)=\{\begin{array}{cc} \frac{f_{\text{ABA}}\left(S_{\text{NaCl}},t\right)+{\left[\text{ABA}\right]}_{\text{ext}}}{\text{max}f_{\text{ABA}}+{\left[\text{ABA}\right]}_{\text{max}}} & \;if\;t>0,\\ 0 & if\;t\leq 0, \end{array}$$

The intracellular ABA signal *S*_ABA_(*t*), also ranging from 0 to 1, is described as above because the amount of endogenous ABA can increase via two routes (Fig. 2a): ABA is synthesized directly in the presence of the salt stress by the function *f*_ABA_ with a fixed maximum value, or is imported from an exogenous pool located in the cell exterior, the size of which is determined by [ABA]_ext_. We set [ABA]_max_ = 100 µM (Xiong et al. 1999).

In the simplified model, we assume that the amount of ABA internally produced from de novo production is negligible compared with the amount imported from the exterior, such that max *f*_ABA_ < < [ABA]_max_ (Windsor et al. 1992, Ren et al. 2007) such that the *S*_ABA_(*t*) is only dependent on [ABA]_ext_ and [ABA]_max_.

#### Regulation of TF activities

The dynamics of *TF*_1_*(*t*), *TF*_2_*(*t*),*TF*_2_(*t*) and *TF*_2_*(*t*) are governed by the structure of the simplified *RD29A* regulatory network (Fig. 2b), which is described by a set of four differential equations

(3) $$\overset{˙}{TF_{1}}=r_{1}+r_{1}^{\tau }S_{\text{NaCl}}\left(t-\tau \right)+\;d_{-1}TF_{1}^{*}\left(t\right)-\left[d_{1b}+\alpha_{1}S_{\text{NaCl}}\left(t\right)+\;u_{1}+\;\delta_{1}\right]TF_{1}\left(t\right),$$

(4) $$\overset{˙}{TF_{1}^{*}}=[d_{1b}+\alpha_{1}S_{\text{NaCl}}\left(t\right)]TF_{1}\left(t\right)-\left(d_{-1}+\delta_{1}\right)TF_{1}^{*}\left(t\right),$$

(5) $$\overset{˙}{TF_{2}}=r_{2}+r_{2}^{\tau }S_{\text{ABA}}\left(t-\tau \right)+C_{2}+\;d_{-2}TF_{2}^{*}\left(t\right)-\left[d_{2b}+\alpha_{2}S_{\text{ABA}}\left(t\right)+\;u_{2}+\;\delta_{2}\right]TF_{2}\left(t\right),$$

(6) $$\overset{˙}{TF_{2}^{*}}=[d_{2b}+\alpha_{2}S_{\text{ABA}}\left(t\right)]TF_{2}\left(t\right)-\left(d_{-2}+\delta_{2}\right)TF_{2}^{*}\left(t\right),$$

The parameters *r_i_*^τ^, *d_ib_*, α*_i_*, *d_–i_*, *u_i_* and δ*_i_*, represent the rates of biochemical processes such as production, degradation and post-translational modification of TF proteins. The parameter τ represents the time delay for the stress inputs to affect accumulation of inactive *TF_i_* via expression of its genes. A description of the parameters is shown in Table 2.

The function *C*_2_ represents production of AREB proteins triggered by NaCl. We assume *C*_1_ = 0 because ABA is not sufficient to induce DREB2 expression on its own (Liu et al. 1998). We set *C*_2_ = *r_c_*^τ^*S*_NaCl_ (*t* –τ), with *r_c_*^τ^ representing the rate of *TF*_2_ production induced by *S*_NaCl_, since AREB expression is known to be triggered by NaCl (Uno et al. 2000, Fujita et al. 2005).

#### Synthesis of mRNA

Our mathematical model describes temporal changes in *RD29A* transcript abundance. Given the lack of information regarding the kinetics of the molecular processes such as TF–DNA binding, TF–TF interaction and RNAP recruitment, we adopted a simple phenomenological description of transcription by assuming linear transcriptional regulation: the quantity of *RD29A* mRNA transcript at time t is defined as

(7) $$m\left(t\right)=[{\text{DREB2}}_{}^{*}]\left(t\right)+k\left[{\text{AREB}}_{}^{*}\right]\left(t\right)$$

where [DREB2*](*t*) and [AREB*](*t*) represent the concentration of post-translationally activated DREB2 and AREB transcription factors at time *t.* An arbitrary quantity *m*(*t*) describes the activity of the *RD29A* promoter, and is equivalent to a weighted sum of [DREB2*](*t*) and [AREB*](*t*) via a constant *k.* Note that the model does not consider dynamics of the transcriptional processes such as TF–DNA binding, RNAP recruitment and mRNA synthesis by assuming that they occur at a much faster time scale compared with intracellular signal transduction (Hargrove et al. 1991).

Like our experimental data, the model output *M*(*t*) captures the relative increase of transcript abundance induced by the inputs, compared with the basal expression level that occurs when *t* = 0. The output is therefore defined as

(8) $$M\left(t\right)=\;\frac{m\left(t\right)}{m(0)}=TF_{1}^{*}\left(t\right)+\;TF_{2}^{*}\left(t\right),$$

where *TF*_1_*(*t*) and *TF*_2_*(*t*) are the concentrations of active DREB2 and AREB normalized by the concentration of *RD29A* transcript from control. The new state variables, *TF*_1_*(*t*) and *TF*_2_*(*t*), are dimensionless quantities describing the change in relative contribution to total mRNA production from each TF.

### Mathematical definition of cross-input modulation

We define cross-input modulation as a change in a parameter in a pathway by its adjacent input. The effect of cross-input modulation on the eligible signaling processes is implemented in the model by replacing the affected parameter *p_j_*, with either of the two functions, $E\left(p_{j}\right)=p_{j}\left(1+c_{j}^{E}S\text{′}(t-\tau )\right)$ for enhancement and $I\left(p_{j}\right)=p_{j}/(1+c_{j}^{I}S\text{′}\left(t-\tau \right))\;$ for attenuation (*j* = 1,2,…9). The variable *S*′ denotes the non-cognate stress input, which can be either *S*_NaCl_ or *S*_ABA_ depending on which pathway *p_j_* belongs to. Note that cross-input modulation is different from cross-input such as *C*_2_ in Equation 5 in that it cannot directly trigger production of TF. The effect of non-cognate input *S*′ on the targeted parameter is delayed by τ, because the synergistic effect in the experimental data appears most pronounced during the late phase of stress response. This is equivalent to assuming that cross-input modulation affects the expression of the genes responsible for the target signaling process. The parameters *c_j_^E^* and *c_j_^I^* represent the strength of enhancement and attenuation of *p_j_* from the presence of cross-input, respectively. The condition *c_j_^E^*^ ^= *c_j_^I^*^ ^= 0 corresponds to the case where no cross-talk interaction is affecting the parameter *p_j_.*

### Model solution

All model solutions were obtained analytically as described in Supplementary Method S1. Model analysis and parameter fitting were carried out using Matlab Release 2014b.

### Parameter estimation

Whilst the values of several parameters are fixed from the literature or analytical derivation (Supplementary Method S1), the values for the unknown parameters were determined by fitting the model to all of our experimental data using a Monte Carlo Simulated Annealing (MCSA) algorithm. The algorithm seeks a parameter vector, **p**, which leads to the best fit between the model solution and experimental data. The objective function *X*(**p**), for the vector **p**, is defined as

(9) $$X\left(p\right)=\;\underset{S}{\sum }\underset{t}{\sum }{\left(\frac{D_{S}\left(t\right)-M_{S,\;p}(t)}{\sigma_{A}D_{S}(t)}\right)}^{2}+{\left(\frac{\Delta D_{\left(S_{NaCl},S_{ABA}\right)}-\Delta M_{(S_{NaCI},S_{ABA}),\;p}}{\sigma_{\text{B}}\Delta D_{\left(S_{NaCI},S_{ABA}\right)}}\right)}^{2}.$$

The vector **p** consists of 10 parameters for the original model, or 11 parameters for each of 18 system structures, which includes one additional parameter describing the strength of cross-input modulation (*c_j_^E^* or *c_j_^I^*). The first term quantifies goodness of fit of the simulated *RD29A* expression profile, *M_S_*_,__**p**_(*t*) to the experimental mean of *RD29A* fold change expression, *D_S_*(*t*), measured at time *t* (0, 0.5, 1, 2, 3 or 5 h) under treatment condition, *S* = (*S*_NaCl_, 0) (*S*_ABA_, 0) or (*S*_NaCl_, *S*_ABA_). The weighting coefficient σ_A_ associated with each data point *D_s_*(*t*) is assumed to be fixed (σ_A_ = 0.02). The second term evaluates the ability of the model with **p** to reproduce the observed synergistic effect, where $\Delta D_{\left(S_{\text{NaCl}},S_{\text{ABA}}\right)}$ and $\Delta M_{(S_{\text{NaCl}},S_{\text{ABA}}),\;p}$ denote the slope of fold increase between 3 and 5 h observed from the experimental data and model solutions under full-strength combined stress treatment, respectively. The second term was introduced to compensate high costs for fitting to the early phase of expression where there are more data points (0, 0.5, 1, 2 h) than in the late phase of expression (3, 5 h). The weighting coefficient σ_B_ associated with the observed slope is fixed (σ_B_ = 0.1). By setting σ_A_ < σ_B_, we provided more weighting in the cost from fitting to the data points compared with the cost of fitting to the gradient. MCSA optimization of the objective function *X* for each system structure identifies **p**′, which approximates the vector of parameters at the global optimum of the objective function *X.*

### Selection of system structures

A residual sum of squares, *Y*, was calculated for each of the 18 system structures identified (Fig. 4) to evaluate the goodness of fit between the observed *RD29A* expression profile and the optimized model only under combined stress:

(10) $$Y\left(p\prime \right)\;=\underset{t}{\sum }{\left(D_{\left(S_{NaCl},S_{ABA}\right)}\left(t\right)-M_{(S_{NaCl},S_{ABA}),p}(t)\right)}^{2},$$

where *D*_(*s*_NaCl_,*s*__ABA__)_(*t*) represents the observed fold change at time *t* under full-strength combined stress treatment, and *M*_(*s*_NaCl_,*s*__ABA__)_(*t*) the simulated fold change using the parameter set **p**′ identified from optimizing the function *X.* The system structures producing the lowest *Y* when implemented in the model were chosen as the viable structures (Fig. 5; Supplementary Fig. S3).

## Funding

This work was supported by the Engineering Physical Sciences Research Council through a Career Acceleration Fellowship [grant No. EP/G007446/1 to R.J.T.]

## Disclosures

The authors have no conflicts of interest to declare.

## Supplementary Material

Supplementary Data

Supplementary Data

## Abbreviations

ABI: ABA-insensitive
ABRE: ABA-responsive element
ABF: ABRE-bindingfactor
AHG: ABA-hypersenstivie germination
AP2/EREBP: APETALA2/ethylene-responsive binding protein
AREB: ABRE-binding
bZIP: basic leucine zipper
CBF: C-repeat-binding factor
DRE: dehydration-responsive element
DREB: DRE-binding
DRIP: DREB-interacting protein
KEG: KEEP ON GOING
PP2C: protein phosphatase 2C
PYL/PYR: pyrobactin-like/pyrobactin
*RD29A*: Response-to-Dehydration 29A
SnRK: SNF-related kinase
TF: transcription factor

## References

[pcw132-B1] Antoni R. Gonzalez-Guzman M. Rodriguez L. Rodrigues A. Pizzio G.A. Rodriguez P.L. (2012) Selective inhibition of clade A phosphatases type 2C by PYR/PYL/RCAR abscisic acid receptors. Plant Physiol. 158: 970–980. 22198272 10.1104/pp.111.188623 PMC3271782

[pcw132-B2] Atkinson N.J. Lilley C.J. Urwin P.E. (2013) Identification of genes involved in the response of Arabidopsis thaliana to simultaneous biotic and abiotic stresses. Plant Physiol. 162: 2028–2041. 23800991 10.1104/pp.113.222372 PMC3729780

[pcw132-B3] Beguerisse-Diaz M. Hernández-Gómez M.C. Lizzul A.M. Barahona M. Desikan R. (2012) Compound stress response in stomatal closure: a mathematical model of ABA and ethylene interaction in guard cells. BMC Syst. Biol. 6: 146. 23176679 10.1186/1752-0509-6-146 PMC3564773

[pcw132-B4] Biswas D. Jiang G. (2011) Differential drought-induced modulation of ozone tolerance in winter wheat species. J. Exp. Bot. 62: 4153–4162. 21527624 10.1093/jxb/err104 PMC3153674

[pcw132-B5] Burgos A. Szymanski J. Seiwert B. Degenkolbe T. Hannah M.A. Giavalisco P. (2011) Analysis of short-term changes in the Arabidopsis thaliana glycerolipidome in response to temperature and light. Plant J. 66: 656–668. 21309866 10.1111/j.1365-313X.2011.04531.x

[pcw132-B6] Chaves M. Maroco J.P. Pereira J.S. (2003) Understanding plant responses to drought from genes to the whole plant. Funct. Plant Biol. 30: 239–264. 10.1071/FP02076 32689007

[pcw132-B7] Chen Y.T. Liu H. Stone S. Callis J. (2013) ABA and the ubiquitin E3 ligase KEEP on GOING affect proteolysis of the Arabidopsis thaliana transcription factors ABF1 and ABF3. Plant J. 75: 965–976. 23742014 10.1111/tpj.12259 PMC3823012

[pcw132-B8] Dodd A.N. Jakobsen M.K. Baker A.J. Telzerow A. Hou S.W. Laplaze L. (2006) Time of day modulates low-temperature Ca2 + signals in Arabidopsis. Plant J. 48: 962–973. 17227550 10.1111/j.1365-313X.2006.02933.x

[pcw132-B9] Estavillo G.M. Crisp P.A. Pornsiriwong W. Wirtz M. Collinge D. Carrie C. (2011) Evidence for a SAL1–PAP chloroplast retrograde pathway that functions in drought and high light signaling in Arabidopsis. Plant Cell 23: 3992–4012. 22128124 10.1105/tpc.111.091033 PMC3246320

[pcw132-B10] Finkelstein R. (2013) Abscisic acid synthesis and response. Arabidopsis Book 11: e0166. 24273463 10.1199/tab.0166 PMC3833200

[pcw132-B11] Fujita Y. Fujita M. Satoh R. Maruyama K. Parvez M.M. Seki M. (2005) AREB1 is a transcription activator of novel ABRE-dependent ABA signaling that enhances drought stress tolerance in Arabidopsis. Plant Cell 17: 3470–3488. 16284313 10.1105/tpc.105.035659 PMC1315382

[pcw132-B12] Furihata T. Maruyama K. Fujita Y. Umezawa T. Yoshida R. Shinozaki K. (2006) Abscisic acid-dependent multisite phosphorylation regulates the activity of a transcription activator AREB1. Proc. Natl. Acad. Sci. USA 103: 1988–1993. 16446457 10.1073/pnas.0505667103 PMC1413621

[pcw132-B13] Gilmour S.J. Fowler S.G. Thomashow M.F. (2004) Arabidopsis transcriptional activators CBF1, CBF2, and CBF3 have matching functional activities. Plant Mol. Biol. 54: 767–781. 15356394 10.1023/B:PLAN.0000040902.06881.d4

[pcw132-B14] Giraud E. Ho L.H. Clifton R. Carroll A. Estavillo G. Tan Y.F. (2008) The absence of ALTERNATIVE OXIDASE1a in Arabidopsis results in acute sensitivity to combined light and drought stress. Plant Physiol. 147: 595–610. 18424626 10.1104/pp.107.115121 PMC2409015

[pcw132-B15] Hargrove J.L. Hulsey M.G. Beale E.G. (1991) The kinetics of mammalian gene expression. Bioessays 13: 667–674. 1789784 10.1002/bies.950131209

[pcw132-B16] Johnson et al. (2014), Trasnscriptomic analysis of Sorghum bicolor responding to combined heat and drought stress, BMC Genomics, 15: 456. 10.1186/1471-2164-15-456 PMC4070570 24916767

[pcw132-B17] Kilian J. Whitehead D. Horak J. Wanke D. Weinl S. Batistic O. (2007) The AtGenExpress global stress expression data set: protocols, evaluation and model data analysis of UV-B light, drought and cold stress responses. Plant J. 50: 347–363. 17376166 10.1111/j.1365-313X.2007.03052.x

[pcw132-B18] Kim J.S. Mizoi J. Yoshida T. Fujita Y. Nakajima J. Ohori T. (2011) An ABRE promoter sequence is involved in osmotic stress-responsive expression of the DREB2A gene, which encodes a transcription factor regulating drought-inducible genes in Arabidopsis. Plant Cell Physiol. 52: 2136–2146. 22025559 10.1093/pcp/pcr143

[pcw132-B19] Kreps J.A. Wu Y. Chang H.S. Zhu T. Wang X. Harper J.F. (2002) Transcriptome changes for Arabidopsis in response to salt, osmotic, and cold stress. Plant Physiol. 130: 2129–2141. 12481097 10.1104/pp.008532 PMC166725

[pcw132-B20] Liu Q. Kasuga M. Sakuma Y. Abe H. Miura S. Yamaguchi-Shinozaki K. (1998) Two transcription factors, DREB1 and DREB2, with an EREBP/AP2 DNA binding domain separate two cellular signal transduction pathways in drought- and low-temperature-responsive gene expression, respectively, in Arabidopsis. Plant Cell 10: 1391–406. 9707537 10.1105/tpc.10.8.1391 PMC144379

[pcw132-B21] Lynch T. Erickson B.J. Finkelstein R.R. (2012) Direct interactions of ABA-insensitive(ABI)-clade protein phosphatase(PP)2Cs with calcium-dependent protein kinases and ABA response element-binding bZIPs may contribute to turning off ABA response. Plant Mol. Biol. 80: 647–658. 23007729 10.1007/s11103-012-9973-3

[pcw132-B22] Mahalingam R. (2015) Consideration of combined stress: a crucial paradigm for improving multiple stress tolerance in plants. InCombined Stress in Plants: Physiological, Molecular and Biochemical Aspects. Edited by Mahalingam R. pp. 1–25. Springer International.

[pcw132-B23] Mishra S. Shukla A. Upadhyay S. Sanchita Sharma P. Singh S. (2014) Identification, occurrence, and validation of DRE and ABRE cis-regulatory motifs in the promoter regions of genes of Arabidopsis thaliana. J. Integr. Plant Biol. 56: 388–399. 24581225 10.1111/jipb.12149

[pcw132-B24] Mittler R. (2006) Abiotic stress, the field environment and stress combination. Trends Plant Sci. 11: 15–19. 16359910 10.1016/j.tplants.2005.11.002

[pcw132-B25] Morimoto K. Mizoi J. Qin F. Kim J.S. Sato H. Osakabe Y. (2013) Stabilization of Arabidopsis DREB2A is required but not sufficient for the induction of target genes under conditions of stress. PLoS One 8: e80457. 24376497 10.1371/journal.pone.0080457 PMC3871162

[pcw132-B26] Msanne et al. (2011), Characterization of abiotic stress-responsive Arabidopsis thaliana RD29A and RD29B genes and evaluation of trangenes, Planta, 234: 97–107. 10.1007/s00425-011-1387-y 21374086

[pcw132-B27] Nakashima K. Ito Y. Yamaguchi-Shinozaki K. (2009) Transcriptional regulatory networks in response to abiotic stresses in Arabidopsis and grasses. Plant Physiol. 149: 88–95. 19126699 10.1104/pp.108.129791 PMC2613698

[pcw132-B28] Nakashima K. Shinwari Z.K. Sakuma Y. Seki M. Miura S. Shinozaki K. (2000) Organization and expression of two Arabidopsis DREB2 genes encoding DRE-binding proteins involved in dehydration- and high-salinity-responsive gene expression. Plant Mol. Biol. 42: 657–665. 10809011 10.1023/a:1006321900483

[pcw132-B29] Pääkkönen J. Vahala J. Pohjola M. Holopainen Y. Kärenlampi L. (1998) Physiological, stomatal and ultrastructural ozone responses in birch (Betula pendula Roth.) are modified by water stress. Plant Cell Environ. 21: 671–684.

[pcw132-B30] Park S.-Y. Fung P. Nishimura N. Jensen D.R. Fujii H. Zhao Y. (2009) Abscisic acid inhibits type 2C protein phosphatases via the PYR/PYL family of START proteins. Science 324: 1068–1071. 19407142 10.1126/science.1173041 PMC2827199

[pcw132-B31] Prasch C.M. Sonnewald U. (2015) Signaling events in plants: stress factors in combination change the picture. Environ. Exp. Bot. 114: 4–14.

[pcw132-B32] Pratt J. et al. (2002), Dynamics of protein turnover, a missing dimension in proteomics, Molecular & Cellular Proteomics, 1: 579–591. 10.1074/mcp.m200046-mcp200 12376573

[pcw132-B33] Puranik S. Sahu P.P. Srivastava P.S. Prasad M. (2012) NAC proteins: regulation and role in stress tolerance. Trends Plant Sci. 17: 369–381. 22445067 10.1016/j.tplants.2012.02.004

[pcw132-B34] Qin F. Sakuma Y. Tran L.S. Maruyama K. Kidokoro S. Fujita Y. (2008) Arabidopsis DREB2A-interacting proteins function as RING E3 ligases and negatively regulate plant drought stress-responsive gene expression. Plant Cell 20: 1693–1707. 18552202 10.1105/tpc.107.057380 PMC2483357

[pcw132-B35] Rasmussen S. Barah P. Suarez-Rodriguez M.C. Bressendorff S. Friis P. Costantino P. (2013) Transcriptome responses to combinations of stresses in Arabidopsis. Plant Physiol. 161: 1783–1794. 23447525 10.1104/pp.112.210773 PMC3613455

[pcw132-B36] Ren H. Gao Z. Chen L. Wei K. Liu J. Fan Y. (2007) Dynamic analysis of ABA accumulation in relation to the rate of ABA catabolism in maize tissues under water deficit. J. Exp. Bot. 58: 211–219. 16982652 10.1093/jxb/erl117

[pcw132-B37] Rizhsky L. Liang H. Shuman J. Shulaev V. Davletova S. Mittler R. (2004) When defense pathways collide. The response of Arabidopsis to a combination of drought and heat stress. Plant Physiol. 134: 1683–1696. 15047901 10.1104/pp.103.033431 PMC419842

[pcw132-B38] Sakuma Y. Maruyama K. Osakabe Y. Qin F. Seki M. Shinozaki K. (2006) Functional analysis of an Arabidopsis transcription factor, DREB2A, involved in drought-responsive gene expression. Plant Cell 18: 1292–1309. 16617101 10.1105/tpc.105.035881 PMC1456870

[pcw132-B39] Suzuki et al. (2014), Abiotic and biotic stress combinations, New Phyologist, 203: 32–43. 10.1111/nph.12797 24720847

[pcw132-B40] Uno Y. Furihata T. Abe H. Yoshida R. Shinozaki K. Yamaguchi-Shinozaki K. (2000) Arabidopsis basic leucine zipper transcription factors involved in an abscisic acid-dependent signal transduction pathway under drought and high-salinity conditions. Proc. Natl. Acad. Sci. 97: 11632–11637. 11005831 10.1073/pnas.190309197 PMC17252

[pcw132-B41] Vile D. Pervent M. Belluau M. Vasseur F. Bresson J. Muller B. (2012) Arabidopsis growth under prolonged high temperature and water deficit: independent or interactive effects? Plant Cell Environ. 35: 702–718. 21988660 10.1111/j.1365-3040.2011.02445.x

[pcw132-B42] Windsor M.L. Milborrow B.V. McFarlane I.J. (1992) Uptake of (+)-S- and (–)-R-abscisic acid by suspension culture cells of Hopbush (Dodonaea viscosa). Plant Physiol. 100: 54–62. 16652999 10.1104/pp.100.1.54 PMC1075516

[pcw132-B43] Xiong L. Ishitani M. Zhu J.K. (1999) Interaction of osmotic stress, temperature, and abscisic acid in the regulation of gene expression in Arabidopsis. Plant Physiol. 119: 205–212. 9880362 10.1104/pp.119.1.205 PMC32221

[pcw132-B44] Xiong L. Zhu J. (2003) Regulation of abscisic acid biosynthesis. Plant Physiol. 133: 29–36. 12970472 10.1104/pp.103.025395 PMC523868

[pcw132-B45] Yamaguchi-Shinozaki K. & Shinozaki K. (1993), Characterization of the expression of a desiccation-responsive rd29 gene of Arabidopsis thaliana and analysis of its promoter in transgenic plants, Molecular & General Genetics, 236: 331–340. 10.1007/BF00277130 8437577

[pcw132-B46] Yoshida T. Fujita Y. Maruyama K. Mogami J. Todaka D. Shinozaki K. (2014) Four Arabidopsis AREB/ABF transcription factors function predominantly in gene expression downstream of SnRK2 kinases in abscisic acid signalling in response to osmotic stress. Plant Cell Environ. 38: 35–49. 24738645 10.1111/pce.12351 PMC4302978

[pcw132-B47] Zhu Y. Qian W. Hua J. (2010) Temperature modulates plant defense responses through NB-LRR proteins. PLoS Pathogens 6: e1000844. 20368979 10.1371/journal.ppat.1000844 PMC2848567

