# Supplementary material for: Synergistic Activation of RD29A Via Integration of Salinity Stress and Abscisic Acid in Arabidopsis thaliana
Source: Plant Cell Physiol. 2016 Aug 6;57(10):2147–60. doi: 10.1093/pcp/pcw132 (PMC5434669; doi:10.1093/pcp/pcw132)
Supplement: Supplementary Data [file pcw132_Supplementary_Data.zip › pcp-2016-e-00320-File008.pdf]

Supporting materials

Synergistic Activation of *RD29A* via Integration of Salinity stress and Abscisic acid in *Arabidopsis thaliana*

Sang Y. Lee, Neville J. Boon, Alex Webb, Reiko J. Tanaka

Figure S1 - *RD29A* expression dynamics under single NaCl, single ABA, and combined NaCl and ABA treatment at equal strength without circadian effect removed.

Figure S2 - *RD29A* expression dynamics under combined NaCl and ABA treatment at unequal strength without circadian effect removed

Figure S3 – Model solutions from the system structures that can reproduce the synergistic effect

Figure S4 – Model solutions from the system structures that cannot reproduce the synergistic effect

Method S1 – Analytical solutions for the model equations

Table S1: Comparison of normalised *RD29A* expression level between 3 and 5 hours of NaCl and ABA treatment (two-sample *t* test)

Table S2 – Parameter values

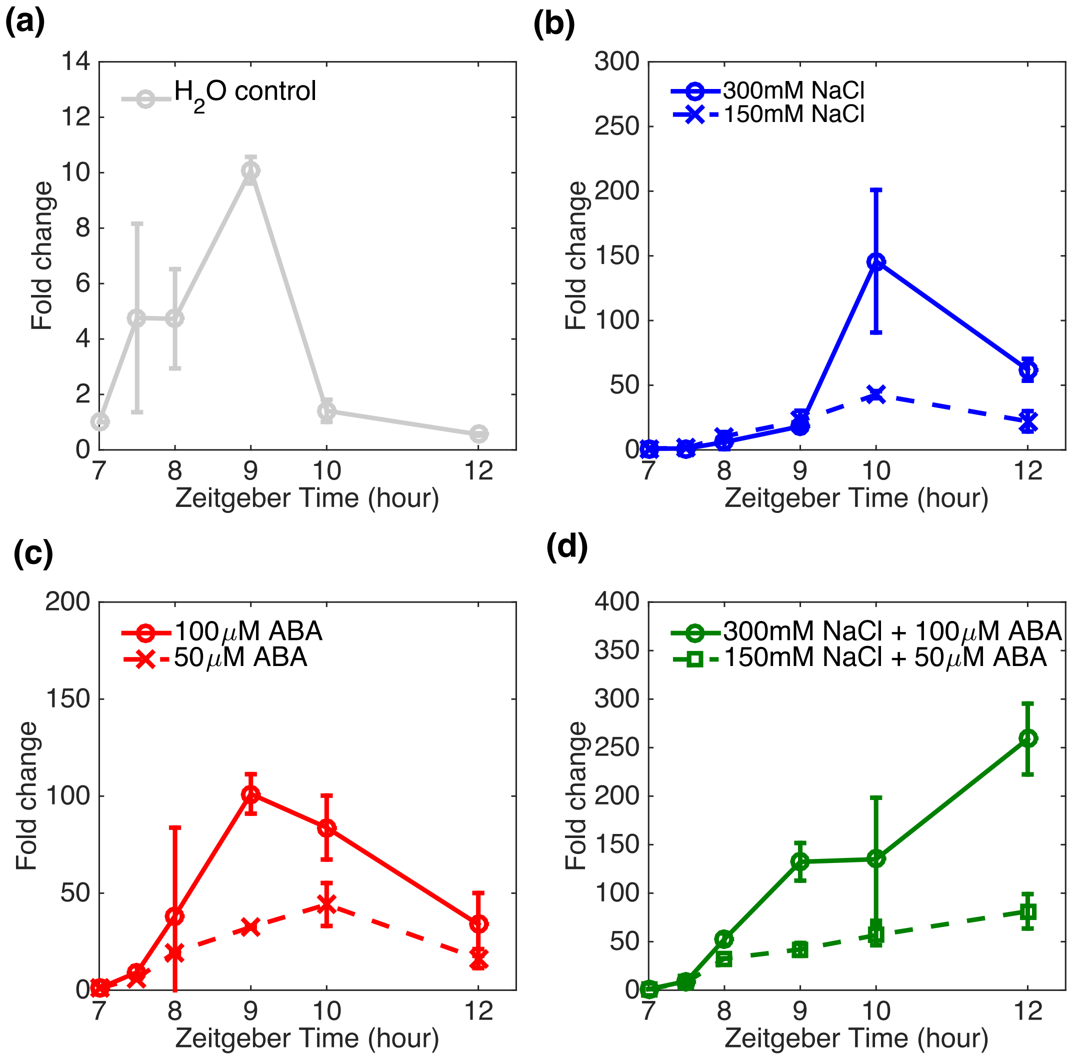

Figure S1 - *RD29A* expression dynamics under single NaCl, single ABA, and combined NaCl and ABA treatment at equal strength without circadian effect removed.

RD29A expression fold changes at different durations of treatment were measured from the samples containing a) only H_2O (control), b) only NaCl (300mM NaCl or 150mM NaCl), c) only ABA (100μM ABA or 50μM ABA), and d) combination of the two inputs either at full-strength or half-strength (300mM NaCl & 100μM ABA or 150mM NaCl & 100μM ABA). The time course profiles induced by single NaCl and ABA indicate that there is distinct change in *RD29A* expression between 9 and 10 Zeitgeber time (2-3 hours after initial treatment).

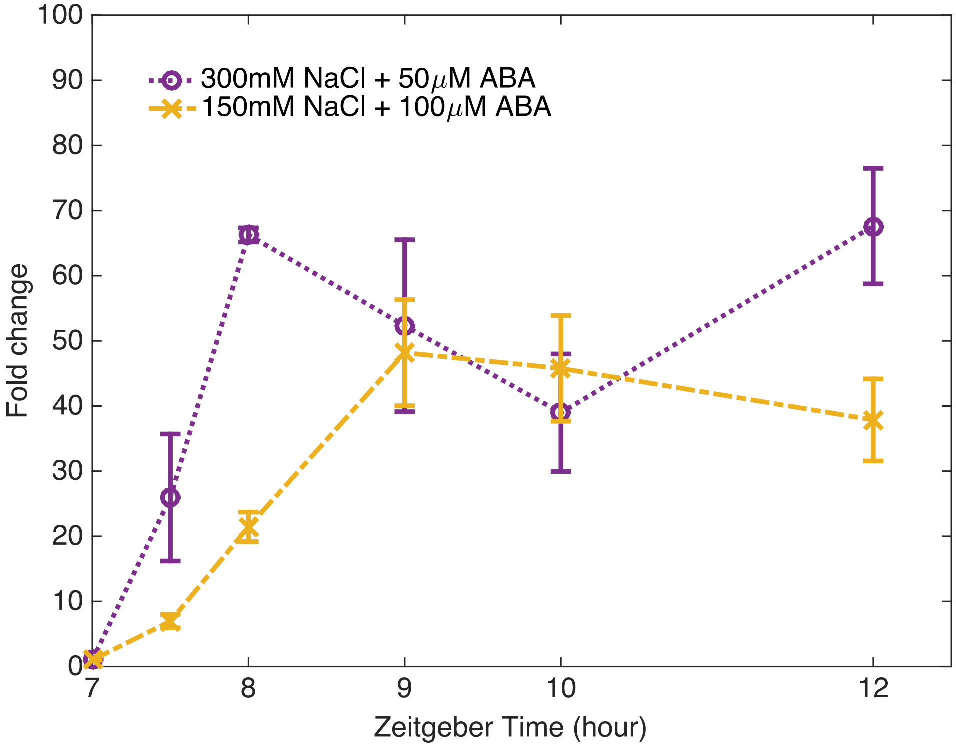

Figure S2 - *RD29A* expression dynamics under combined NaCl and ABA treatment at unequal strength without circadian effect removed

RD29A expression dynamics was measured again to validate the predicted responses to the combination of the two inputs at unequal strengths (150mM NaCl & 100μM ABA or 300mM NaCl & 50μM ABA).

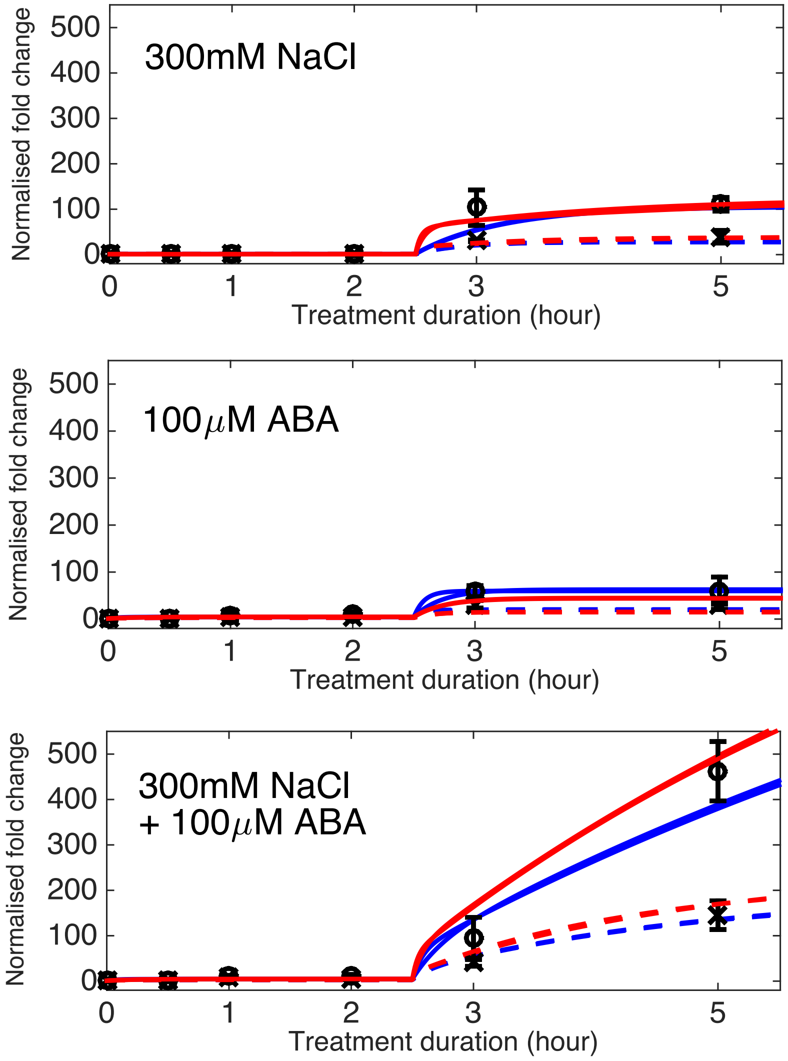

Figure S3 – Model solutions from the system structures that can reproduce the synergistic effect

Comparison between model solutions from the remaining four system structures out of the five in total with the experimental data. The result for $I(d_{-1})$ is provided in Figure 5 of the main text. Solid and dashed lines represent simulated profiles induced by stresses at full and half-strength, respectively. Each plot contains two sets of red lines showing the model solutions from $I(u_2)$ and $I(d_{-2})$, and two sets of blue lines showing the solutions from $I(u_1)$ and $E(\alpha_1)$. Overall, the model solutions from all four structures provide reasonable qualitative description of the behaviours observed from *RD29A* expression measured under all treatment conditions.

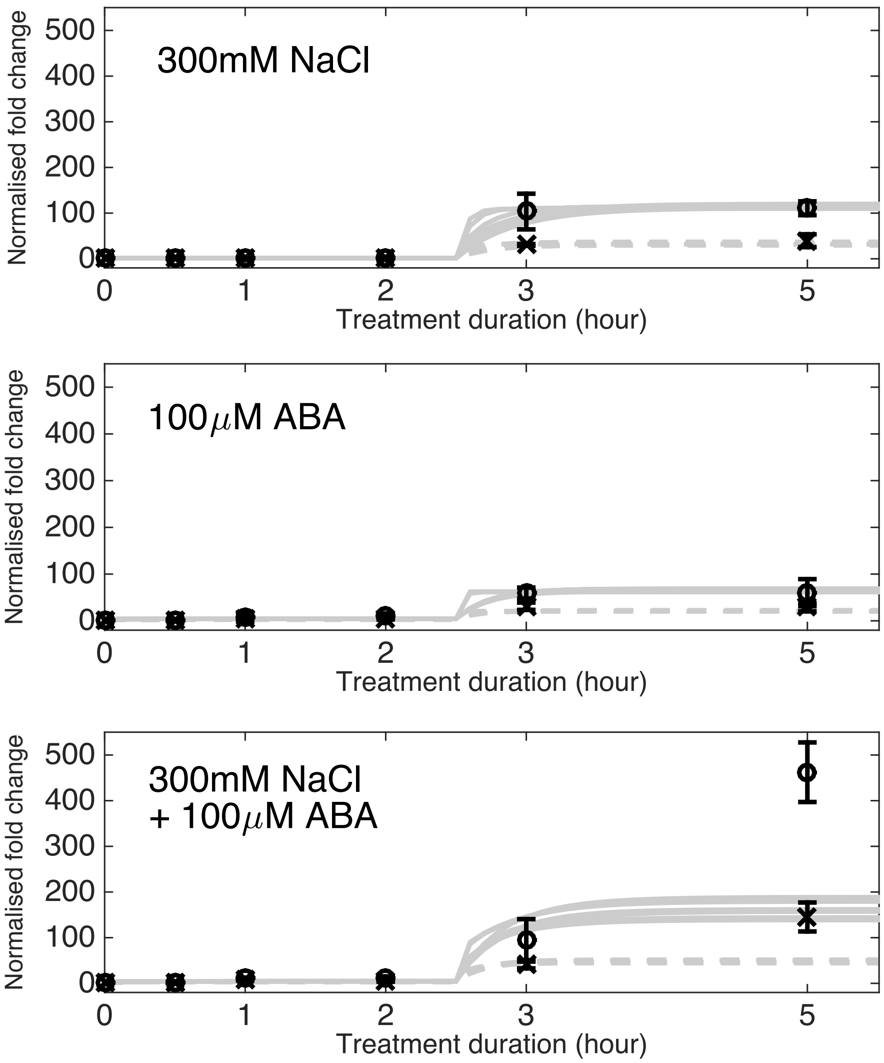

Figure S4 – Model solutions from the system structures that cannot reproduce the synergistic effect

Model solutions from the remaining 13 system structures after parameter optimisation (grey lines) compared with the experimental data (points). Solid and dashed lines represent simulated profiles induced by treatments at full and half-strength, respectively. The models implementing one of the remaining 13 cross-input modulation cannot reproduce the synergistic effect observed from the experimental data even after parameter optimisation.

Method S1 – Analytical solutions for the model equations

Before stress input ($\boldsymbol{S_1 = S_2 = 0, t \leq 0}$), the system is assumed to be at steady state ($\boldsymbol{\dot{TF}_1 = \dot{TF}_2 = \dot{TF}_1^* = \dot{TF}_2^* = 0}$). Some parameters are fixed based upon the literature (as discussed in the main text) and the steady-state solutions before exposure to stress ($\boldsymbol{t \leq 0}$) can be determined as follows:

$$
\begin{aligned}
TF_1(t \leq 0) &= 0, \\
TF_1^*(t \leq 0) &= 0, \\
TF_2(t \leq 0) &= \frac{(\delta_2 + d_{-2})}{d_{2b}}, \\
TF_2^*(t \leq 0) &= 1.
\end{aligned}
$$

After exposure to stress ($t > 0$) we solve our ordinary differential equations by first rewriting in the form

$$
\begin{aligned}
\dot{TF}_i &= \Theta_i - (\Delta_i + A_i)TF_i + d_{-i}TF_i^* , \\
\dot{TF}_i^* &= A_i TF_i - (\delta_i + d_{-i})TF_i^*,
\end{aligned}
$$

with i = 1, 2 representing DREB2 and AREB pathway, respectively, where $\Theta_1 = r_1^\tau S_1(t - \tau) + C_1$, $\Theta_2 = r_2 + r_2^\tau S_2(t - \tau) + C_2$, $\Delta_1 = u_1 + \delta_1$, $\Delta_2 = u_2 + \delta_2$, $A_2 = \alpha_1 S_1(t)$, and $A_2 = d_{2b} + \alpha_2 S_2(t)$.

Noting that Θ_i and A_i are constant over our defined time windows, the solutions are

$$
\begin{aligned}
TF_i(t) &= K_{2,i}(t)\exp\big(\lambda_{i+}T(t)\big) + K_{1,i}(t)\exp\big(\lambda_{i-}T(t)\big) + \frac{\Theta_i(\delta_i + d_{-i})}{(A_i + \Delta_i)\delta_i + d_{-i}\Delta_i}, \\
TF_i^*(t) &= \frac{1}{d_{-i}}\left[K_{2,i}(t)(\lambda_{i+} + \Delta_i + A_i)\exp\big(\lambda_{i+}T(t)\big)\right] \\
&\quad + \frac{1}{d_{-i}}\left[K_{1,i}(t)(\lambda_{i-} + \Delta_i + A_i)\exp\big(\lambda_{i-}T(t)\big) - \Theta_i + \frac{(A_i + \Delta_i)\Theta_i(\delta_i + d_{-i})}{(A_i + \Delta_i)\delta_i + d_{-i}\Delta_i}\right],
\end{aligned}
$$

where the eigenvalues of the system are

$$
\lambda_{i\pm} = \frac{1}{2}\left[-(A_i + \Delta_i + d_{-i} + \delta_i) \pm \sqrt{(A_i + \Delta_i + d_{-i} + \delta_i)^2 - 4(A_i\delta_i + d_{-i}\Delta_i + \delta_i\Delta_i)}\right]
$$

and

$$
\begin{aligned}
K_{1,i}(t) = \frac{1}{\lambda_{i-} - \lambda_{i+}}\Big(& d_{-i}TF_{i,ss}^* + \Theta_i - (\lambda_{i+} + \Delta_i + A_i)TF_{i,ss}(t \leq 0) \\
& + \lambda_{i+}\frac{\Theta_i(\delta_i + d_{-i})}{(A_i + \Delta_i)\delta_i + d_{-i}\Delta_i}\Big),
\end{aligned}
$$

$$K_{2,i}(t) = TF_{i,ss} - K_{1,i} - \frac{\Theta_i(\delta_i + d_{-i})}{(A_i + \Delta_i)\delta_i + d_{-i}\Delta_i},$$

with

$$TF^*_{i,ss} = \begin{cases} TF^*_i(0) & \text{if } 0 < t \le \tau, \\ TF^*_i(\tau) & \text{if } \tau < t, \end{cases}$$

$$TF_{i,ss} = \begin{cases} TF_i(0) & \text{if } 0 < t \le \tau, \\ TF_i(\tau) & \text{if } \tau < t, \end{cases}$$

and

$$T(\mathrm{t}) = \begin{cases} t & \text{if } 0 < t \le \tau, \\ t - \tau & \text{if } \tau < t. \end{cases}$$

Note that the value of Θ_i, Δ_i and A_i can change between the defined time windows T if the associated parameters are affected by cross-input modulation, as the affected parameter p_j is replaced by either $E(p_j)$ or $I(p_j)$.

- Fixing a parameter based on steady-state assumption

The assumption that the system is at steady state before introduction of stress enables further reduction of parameter space. From the assumption that the system is at steady state before treatment,

$$M(t \le 0) = TF^*_2(t \le 0) = 1.$$

The steady-state solution for TF^*_2 at $t \le 0$ can be expressed in terms of model parameters by setting $\dot{TF}^*_2 = 0$, which leads to

$$\frac{d_{2b} r_2}{[\delta_2^2 + \delta_2(d_{-2} + d_{2b}) + u_2 d_{-2}]} = 1.$$

Rearranging above enables determination of a parameter in terms of other parameters, thus fixing the value of the chosen parameter. Here, we fix r_2 in terms of δ_2, d_{2b}, d_{-2} and u_2:

$$r_2 = \frac{1}{d_{2b}}[\delta_2^2 + \delta_2(d_{-2} + d_{2b}) + u_2 d_{-2}].$$

Table S1: Comparison of normalised *RD29A* expression level between 3 and 5 hours of NaCl and ABA treatment (two-sample *t* test)

Treatment condition	Mean normalised expression level (standard deviation)		p-value
	3 hour	5 hour	
300mM NaCl	103.4 (39.1)	110.7 (15.1)	0.825
150mM NaCl	30.3 (1.92)	39.8 (14.1)	0.605
100µM ABA	59.4 (11.7)	60.3 (28.9)	0.969
50µM ABA	31.3 (7.84)	29.8 (8.73)	0.789
300mM NaCl + 100µM ABA	95.7 (44.9)	462.3 (65.2)	0.007**
150mM NaCl + 50µM ABA	40.2 (7.44)	145.3 (31.7)	0.005**

** Significant difference at $p < 0.01$

Table S2 – Parameter values

- *Fixed parameters* (for all model simulations)

Parameter	r_1	d_{1b}	δ_1	δ_2	τ
Value	0	0	0.02	0.02	2.5

The length of time delay is fixed ($\tau = 2.5\,h$) based upon the observations from our experimental data that the abrupt increases in transcript abundance occur between 2 to 3 hours of stress treatment. The natural degradation rates of both DREB2 and AREB proteins are fixed ($\delta_1 = \delta_2 = 0.02\,h^{-1}$) according to the proteome-wide *in vitro* measurements of yeast proteins (Pratt et al., 2002). Due to absence of basal DREB2 gene expression in unstressed conditions (Liu et al., 1998), the basal production rate of DREB2 protein is assumed to be negligible ($r_1 = 0\,h^{-1}$). Because overexpression of DREB2 gene without stress cue does not lead to expression of the DREB2 target genes at downstream (Sakuma et al., 2006), the basal post-translational activation rate of DREB2 is also assumed to be negligible ($d_{1b} = 0\,h^{-1}$).

- *Estimated parameters* (4 s.f.)

All parameters in the table below are in the unit of h^{-1}.

Model type	Initial	$I(d_{-1})$	$I(u_1)$	$E(\alpha_1)$	$I(d_{-2})$	$I(u_2)$	$I(\alpha_1)$	$I(\alpha_2)$	$I(r_c^\tau)$	$I(r_1^\tau)$
Corresponding Figure(s)	Fig.3	Fig.5, 6	Fig.S3			Fig.6,S3	Fig.S4			
r_1^τ	843.9	270.7	153.3	147.7	2333	1033	1294	251.4	183.1	502.7
α_1	762.4	617.5	4462	6303	1119	1205	1397	5534	7366	3754
d_{-1}	24.34	3.233	135.0	35.82	62.47	67.49	35.86	70.08	59.57	117.4
u_1	334.2	492.7	48.61	245.3	744.8	286.5	526.2	221.6	277.9	160.5
r_c^τ	411.8	848.6	781.4	361.2	449.2	317.9	473.2	530.1	441.6	475.0
r_2	20.61	17.32	9.504	7.745	43.16	2.561	147.9	195.3	186.5	136.2
d_{2b}	311.3	267.7	269.9	807.9	173.4	222.9	301.8	298.7	298.7	302.0
r_2^τ	1346	3120	2923	1254	1687	1153	1769	1943	1622	1768
α_2	95.29	171.1	236.7	288.4	50.76	552.6	77.96	119.4	119.9	78.66
d_{-2}	88.97	107.9	72.37	81.69	185.2	14.71	135.0	97.90	80.30	135.0
u_2	549.0	42.89	35.35	76.38	40.37	38.47	330.5	595.7	693.5	304.7
c^E				11.81						
c^I		136.4	12.37		23.07	27.24	40.76	17.37	16.79	13.74
X	7.188x10^4	6.338 x10^4	6.386x10^4	6.489x10^4	6.525x10^4	6.523 x10^4	6.499x10^4	6.437x10^4	6.431x10^4	6.492x10^4
Y	1.437x10^5	2.761 x10^3	7.023x10^3	8.586x10^3	5.431x10^3	6.558 x10^3	1.042x10^5	1.051x10^5	9.398x10^4	1.054x10^5

Model type	$I(r_2^\tau)$	$E(\alpha_2)$	$E(d_{-1})$	$E(d_{-2})$	$E(r_c^\tau)$	$E(r_1^\tau)$	$E(r_2^\tau)$	$E(u_1)$	$E(u_2)$
Corresponding figure	Fig.S4								
r_1^τ	301.2	265.0	1716	380.6	322.8	270.3	340.6	479.6	375.9
α_1	5684	5662	4781	1755	1178	4780	1491	6957	1262
d_{-1}	83.20	95.87	202.2	21.05	23.56	94.55	11.92	102.1	30.03
u_1	185.5	144.3	429.7	278.4	138.3	120.5	370.9	330.6	142.0
r_c^τ	646.1	353.1	408.5	234.4	250.4	359.0	146.7	467.9	283.5
r_2	70.2	17.79	91.02	205.8	0.8459	8.214	11.85	178.4	503.8
d_{2b}	298.2	278.2	301.0	319.8	8184	2150	3874	298.7	307.3
r_2^τ	2344	1289	1523	880.8	844.9	1171	501.25	1717	1052
α_2	130.5	83.93	106.4	122.4	821.2	508.2	193.4	65.55	64.27
d_{-2}	106.8	92.89	85.73	45.60	187.5	111.2	227.36	158.2	100.6
u_2	195.8	53.21	319.5	1443	36.03	158.4	201.5	336.7	1539
c^E		0.1243	24.60	10.48	2.230	0.021	0.047	39.65	11.51
c^I	10.30								
X	$6.413\text{x}10^4$	$6.477\text{x}10^4$	$6.501\text{x}10^4$	$6.522\text{x}10^4$	$6.562\text{x}10^4$	$6.484\text{x}10^4$	$6.602\text{x}10^4$	$6.453\text{x}10^4$	$6.472\text{x}10^4$
Y	$9.131\text{x}10^4$	$7.752\text{x}10^4$	$1.054\text{x}10^5$	$1.018\text{x}10^5$	$8.291\text{x}10^4$	$8.165\text{x}10^4$	$8.199\text{x}10^4$	$1.047\text{x}10^5$	$1.036\text{x}10^5$

References for the fixed parameters

1. Pratt, J. M. *et al.* Dynamics of protein turnover, a missing dimension in proteomics. *Mol. Cell. Proteomics* **1,** 579–591 (2002).

2. Liu, Q. *et al.* Two transcription factors, DREB1 and DREB2, with an EREBP/AP2 DNA binding domain separate two cellular signal transduction pathways in drought- and low-temperature-responsive gene expression, respectively, in Arabidopsis. *Plant Cell* **10,** 1391–406 (1998).

3. Sakuma, Y. *et al.* Functional Analysis of an Arabidopsis Transcription Factor , DREB2A , Involved in Drought-Responsive Gene Expression. **18,** 1292–1309 (2006).
