# Supplementary material for: Synergistic Activation of RD29A Via Integration of Salinity Stress and Abscisic Acid in Arabidopsis thaliana
Source: Plant Cell Physiol. 2016 Aug 6;57(10):2147–60. doi: 10.1093/pcp/pcw132 (PMC5434669; doi:10.1093/pcp/pcw132)
Supplement: Supplementary Data [file supp_57_10_2147__index.html]

Synergistic Activation of RD29A Via Integration of Salinity Stress and Abscisic Acid in Arabidopsis thaliana — Synergistic Activation of RD29A Via Integration of Salinity Stress and Abscisic Acid in Arabidopsis thaliana — Supplementary Data 

# Synergistic Activation of *RD29A* Via Integration of Salinity Stress and Abscisic Acid in *Arabidopsis thaliana*

## Supplementary Data

files

- Supplementary Data - pdf file
